# *Scaphochlamys disticha* (Zingiberaceae), a new species with distichous inflorescence from Peninsular Malaysia

**DOI:** 10.3897/phytokeys.99.22287

**Published:** 2018-05-16

**Authors:** Yen Yen Sam, Halijah Ibrahim

**Affiliations:** 1 Forest Research Institute Malaysia, 52109 Kepong, Selangor, Malaysia; 2 Institute of Biological Sciences, Faculty of Science, University of Malaya, 50603 Kuala Lumpur, Malaysia

**Keywords:** A new species of ginger, *Scaphochlamys disticha* Y.Y.Sam & H.Ibrahim, sp. nov., from Terengganu, Peninsular Malaysia is described and illustrated; colour plates and conservation status are also provided. The species is characterised by its large inflorescence with distichously arranged floral bracts. Ginger, flora, taxonomy, Terengganu

## Introduction

Peninsular Malaysia has a very rich and diverse flora with approximately 8,300 species of vascular plants (Saw and Chung 2015).The flora of Peninsular Malaysia is well documented and studied compared to other floristic sites within the region (Ridley 1922, 1923, 1924, 1924a, 1925; Turner 1995; Whitmore 1972–1973; Ng 1978, 1989). Nevertheless, the ongoing Flora of Peninsular Malaysia project continues to uncover new species through extensive collection, systematic documentation and taxonomic study (Kiew et al. 2010, 2011, 2012, 2013, 2015, 2017; Parris et al. 2010, 2013). *Scaphochlamys disticha* is the latest addition to the Flora. *Scaphochlamys* Baker (1892) is a common understorey plant in the lowland forest of Peninsular Malaysia; 28 species have been documented thus far (Holttum 1950; Sam and Saw 2005; Sam et al. 2010, 2015). The state of Terengganu is floristically rich and diverse with 18 species recorded; nine including the new species are endemic to the state.

## Taxonomy

### Scaphochlamys disticha

Taxon classificationPlantaeZingiberalesZingiberaceae

Y.Y.Sam & H.Ibrahim sp. nov.

urn:lsid:ipni.org:names:60476475-2

Figures 1A–G , 2

#### Type.

MALAYSIA. Peninsular Malaysia, Terengganu, Ulu Terengganu Tambahan Forest Reserve, 4°57.99'N, 102°56.91'E, 237m a.s.l., 1 March 2016, Sam et al. FRI 69123 (holotype KEP!; isotype AAU, BKF!, E!, K!, KLU!, SAN!, SING!).

#### Diagnosis.

Similar to S. klossii Holttum var. klossii by its ascending rhizomes supported by fine stilt roots, leafy shoots with multiple leaves, successive shoots emerging within the leaf axil, long leaf sheath with broad and thin edges and elliptic leaf blades. The most distinct feature of *S. disticha* is its distichous floral bracts which are easily recognised from the spirally arranged bracts in S. klossii var. klossii. Other morphological characteristics which can be used to separate *S. disticha* are the spathulate bracts versus involute bracts observed in S. klossii var. klossii and smaller flowers (35–40 mm long vs. 42–50 mm long). The thick woolly hairs covering the sheath, petiole and inflorescence in S. klossii var. klossii are absent from *S. disticha*.

**Figure 1.**
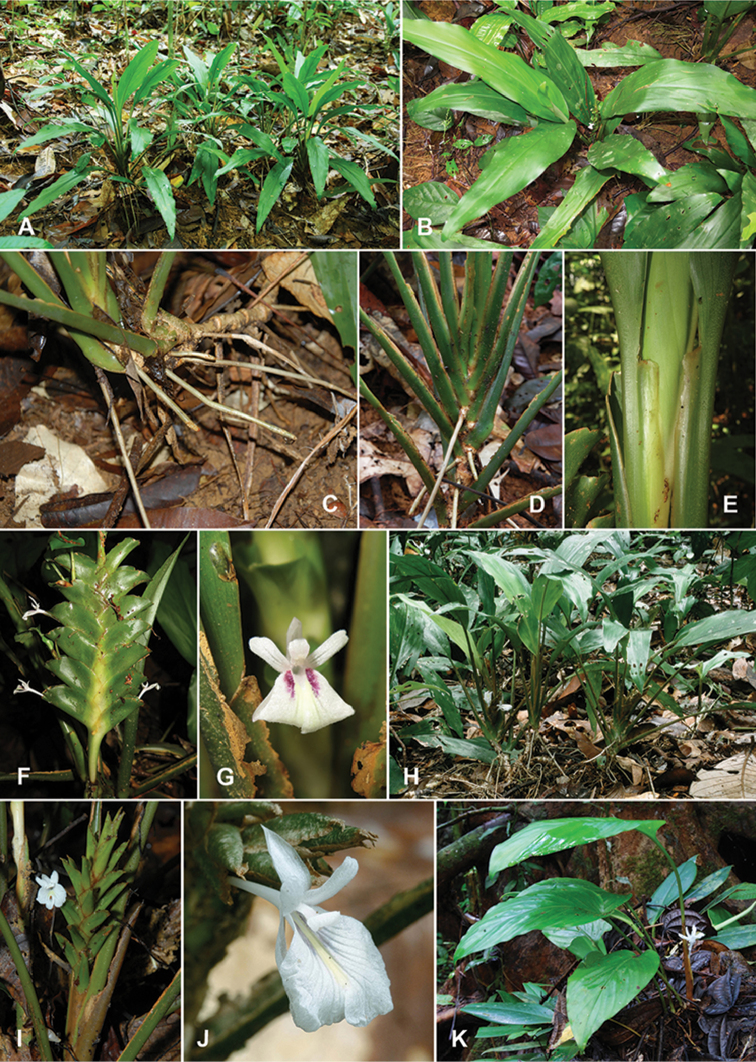
**A–G** *Scaphochlamys disticha*: **A** Habit **B** Leafy shoots close together **C** Rhizome and stilt roots **D** Distichous leaf sheaths **E** Thin and broad margin of leaf sheath **F** Inflorescence **G** Flower **H–J** S. klossii var. klossii **H** Habit **I** *Inflorescence* **J** Flower **K** *S. calcicola*. Photographs **A, C–D, H–K** by YY Sam; **B, E–G** by K Imin.

#### Description.

Terrestrial herb, evergreen, 30–70 cm tall. Rhizome ascending above ground, supp glabrous orted by fine aerial roots, turning upright at apex; successive shoots emerging from third or fourth leaf axil. Leafy shoot composed of (2–3)–11 leaves, glabrous; bladeless sheath usually 2, largest 9–16 cm long, green, coriaceous, persistent; leaf sheath 11–15 cm long, green, arranged distichously, margin very thin and broad, drying early; ligule ca. 2 mm long, rounded, membranous; petiole absent, sheath very long and extending to base of lamina; lamina 32–42 × 6–9 cm, narrowly elliptic, base cuneate or attenuate, apex acute, adaxial surface green, abaxial surface lighter green, with very light red tinge when young. Inflorescence terminal, 11–15 cm long, glabrous, green; peduncle 3–4.5 cm long, embedded within leaf sheath; rachis 7.5–11 cm long, consisting of (9–)11–16 floral bracts, distichously arranged, axis completely hidden; floral bracts 30–38 mm long, spathulate, 27–35 mm wide when flattened, almost orbicular, thickly coriaceous, stiff, green, glabrous. First bracteole 16–17 mm long, about half the length of floral bract, broadly ovate, slit to the base, 2-keeled, margin inflexed along the keels, overlapping, glabrous, apex broadly acute; subsequent bracteoles 7–10 mm, broadly ovate-triangular when flattened, thin, 1-keeled, apex acute and mucronate. Flowers 35–40 mm long, white, except labellum; 18–23 flowers in each cincinnus; calyx 9–11 mm long, tubular, glabrous, apex bifid, split ca. 3 mm unilaterally from apex; floral tube 24–28 mm long, ca. 1 mm diameter widening to 2 mm distally, long exserted from floral bract; dorsal corolla lobe 6–8 × 2–3 mm, linear, margin inflexed, apex hooded, ending with short pointed tip, lateral corolla lobes 6–7 × ca. 2 mm, linear, margin inflexed, apex obtuse; staminodes 4–6 × 1.5–2 mm, oblong, apex obtuse, adaxial surface covered with glandular hairs; labellum 8–9 × 7–8 mm, ovate, apex bilobed with ca. 3 mm cleft, apex strongly reflexed, adaxial surface covered with glandular hairs, yellow median band with red purple patches at the base. Stamen ca. 4 mm long, covered with glandular hairs; filament ca. 2 mm long; anther ca. 2.5 mm long, base shortly spurred, anther thecae dehiscing longitudinally; anther-crest ca. 1 mm long, extended and recurved, apex rounded. Ovary cylindrical, ca. 2 mm long, glabrous, unilocular with 3 ovules, placentation free-basal; stigma ca. 1 × 1 mm, funnel-shaped, pubescent, ostiole with a long slit; epigynous glands filiform, two, ca. 2 mm long. Fruit ca. 12 × 6 mm, oblong-ellipsoid, wall fleshy and translucent; seeds 3, ellipsoid, ca. 8 × 3 mm, arillate, aril white and laciniate.

**Figure 2.**
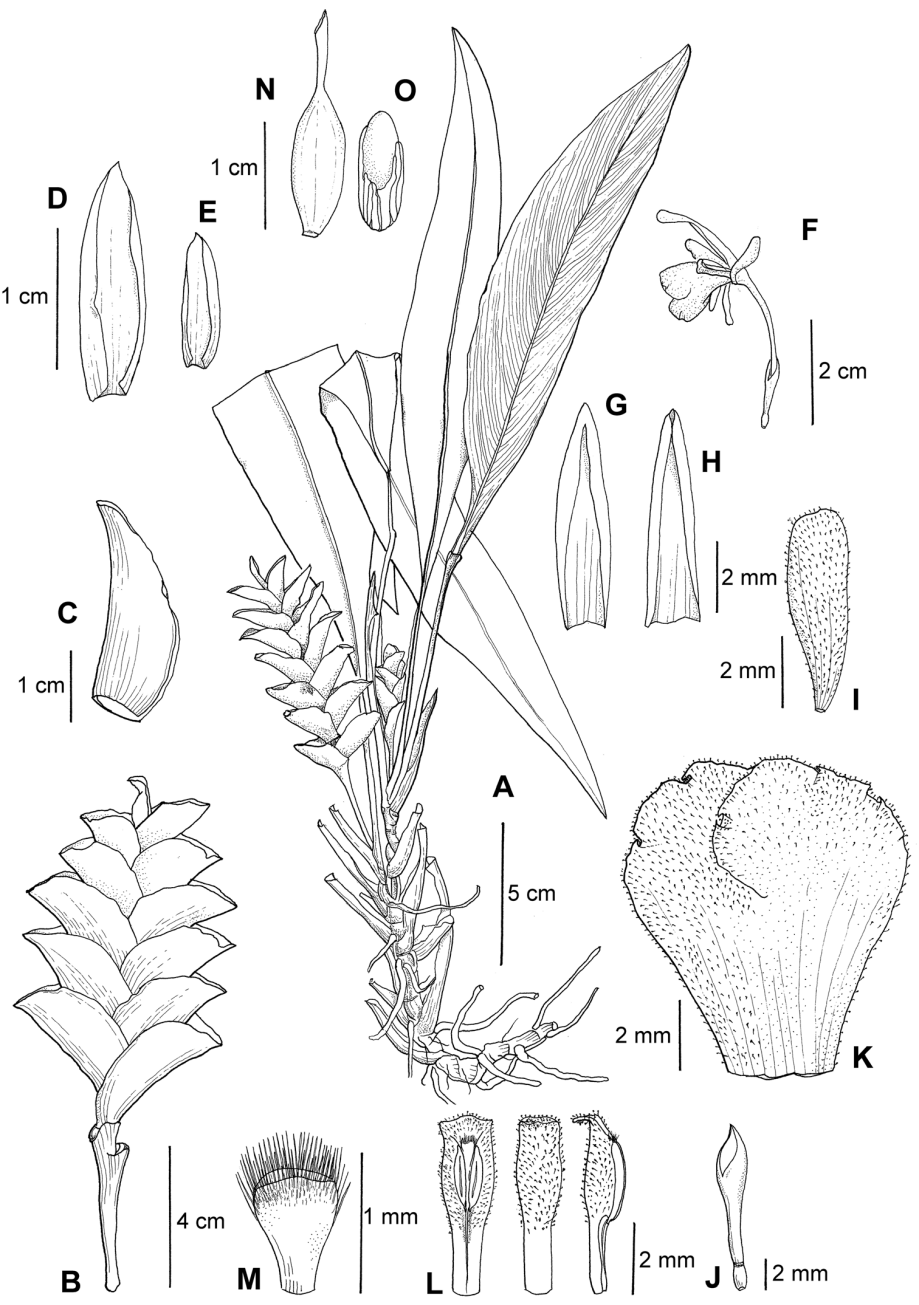
*Scaphochlamys disticha*: **A** Habit **B** Inflorescence **C**Floral bract **D** First bracteole **E** Second bracteole **F** Flower **G** Dorsal corolla lobe **H** Lateral corolla lobe **I** Staminode **J** Ovary and calyx **K** Labellum **L** Stamen **M** Stigma **N** Fruit **O** Seed. Drawn by MN Aidil from Sam et al. FRI 69123.

#### Distribution.

Endemic in Peninsular Malaysia, Terengganu, Ulu Terengganu Tambahan Forest Reserve.

#### Etymology.

The specific epithet is derived from the Latin ‘*disticha*’ referring to the distichously arranged floral bracts.

#### Habitat and ecology.

The plants are found in lowland dipterocarp forest growing abundantly in the bright but shady conditions provided by the canopy openings. A search of the surrounding area found the population to be highly localised, restricted to the mid slope of the valley at Sekayu Waterfall. *Scaphochlamys disticha* flowers gregariously after the northeast monsoon ends in February–March.

#### Conservation status.

Rare (RA). Currently, *Scaphochlamys disticha* is only known from its type locality in Ulu TerengganuTambahan Forest Reserve. The plants are found in the valley of Sekayu Waterfall which is within the reserved forest and this area has been classified as an amenity forest. In the National Forestry Act 1984, amenity forests are managed as protection forests where no logging activity or extraction of other forest produce is allowed. The species is therefore listed as RA following the guidelines in the Malaysian Plant Red List because the species is considered rare but not threatened by extinction (Chua 2012).

#### Other specimen examined.

Peninsular Malaysia. Terengganu, Hutan Lipur Sekayu, 5 May 1986 Kiew s.n. (KEP!)

#### Notes.

*Scaphochlamys disticha* with its fine stilt roots, above ground polyphyllous stem, long leaf sheath, sessile leaves with elliptic blade closely resembles S. klossii var. klossii but it lacks the dense and woolly indumentum found on S. klossii var. klossii. The main difference between these species is the inflorescence structure; *S. disticha* has distichous floral bracts as opposed to the spirally arranged bracts in S. klossii var. klossii (Figure 1J).

Distichously arranged floral bracts are rare in *Scaphochlamys*. *Scaphochlamys calcicola* A.D.Poulsen & R.J.Searle in Borneo is another species reported with distichous bracts (Poulsen and Searle 2005) but a recent collection, Sam FRI 50290 (KEP) from Seromah, Bau, Sarawak showed spiral bracts. This may be an aberrant form; more collections from other sites are required to confirm the typical state. However, the habit and inflorescence structure of *S. calcicola* are noticeably different from *S. disticha* (Figure 1K). *Scaphochlamys calcicola* is a unifoliate plant with long-petiolate leaf. The petiole can measure up to 39 cm long which is clearly distinct from the sessile leaves in *S. disticha*. Another apparent feature is the green spathulate floral bracts of *S. disticha* contrary to the boat-shaped bracts in *S. calcicola*.

Newman (1995) has described *Distichochlamys* M.F.Newman based on its distichous floral bracts. The genus closely resembles *Scaphochlamys* and phylogenetic studies have confirmed both as distinct sister-clades in the family Zingiberaceae (Kress et al. 2002; Ngamriabsakul et al. 2004; Takano and Nagamasu 2007; Sam et al. 2016). There are several characters to distinguish *Distichochlamys* from *Scaphochlamys*, i.e. distichous versus spiral bracts, tubular versus open bracteoles, anthers spurless versus spurred and chromosome number 2n=26 versus 2n=28 (Newman 1995; Larsen and Newman 2001; Poulsen and Searle 2005). The open bracteole of *Scaphochlamys* is most useful in distinguishing it from *Distichochlamys*; the arrangement of bracts and anther spurs are less distinctive. Here, the authors proposed two additional morphological characters to separate the two genera: inflorescence structure and placentation. In *Distichochlamys*, the distichous bracts were held to one side of the rachis and Rehse and Kress (2003) reported 10–30° slant from the vertical axis in *D. rubrostriata* (Newman 1995; Larsen and Newman 2001; Rehse and Kress 2003; Leong-Škorničková pers. comm.). Leong-Škorničková (*pers*. *comm*.) observed the deviation is especially prominent in young inflorescences and becoming less conspicuous in some old inflorescences. On the contrary, the distichous bracts of *S. calcicola* and *S. disticha* are arranged bilaterally on the inflorescence axis. Placentation is another diagnostic character not discussed previously. *Distichochlamys* has trilocular ovary with axile placentation differing from the unilocular ovary with free-basal placentation of *Scaphochlamys*. In the absence of chromosome number and molecular data, the unilateral inflorescence and trilocular ovary with axile placentation further support the position of *S. disticha* in *Scaphochlamys*.

Larsen and Newman (2001) reported a possibly undescribed *Scaphochlamys* species from Peninsular Malaysia with distichous bracts deposited in AAU. However, a recent search in AAU did not find such a specimen (B. Øllgaard, pers. comm.).The AAU specimen may very well represent another undescribed species from Peninsular Malaysia.

## Supplementary Material

XML Treatment for Scaphochlamys disticha
